# Circular

**Published:** 1852-08

**Authors:** 


### CIRCULAR.

We publish below the Circular of the Committee *on the Epidemics of Tennessee and Kentucky*, which we hope will awaken a due interest in the physicians of those States. The chairman of the committee informs us that although the profession was not well prepared to respond to the call of the American Medical Association when made a year ago, still a number of physicians set zealously to work and made out valuable reports. The circumstances are now more favorable. The same committee is to be continued for five years, and those who could not at once make a report will be able to do so at their leisure. But all who have any desire or ambition to aid in the advancement of their profession by investigating the epidemics passing under their observation, should go to work immediately, and collect, and arrange the statistics asked for by the committee. The enterprise is a highly important one, and it is to be expected that our brethren generally will be disposed to expend time and labor in its furtherance.

GEORGETOWN, July 26, 1852.

*To the Editors of the Western Journal of Medicine and Surgery*:

Gentlemen—I send herewith a call upon our brethren for materials for compiling a history of the Epidemics of Kentucky and Tennessee. Will you be so good as to allow it a place in the forthcoming number of your Journal. I hope, too, that you will be able to allow room to the accompanying table. I look upon it as a very important item in the information sought. With all the disadvantages of last year I was very much gratified at the number of cases tabled. There were about 400 of dysentery alone. Under more favorable circumstances I hope we may do still better. I think the table at the end of each disease treated of gives a finished appearance to the job. Besides, it is an example set of giving the relative mortality of each disease, which I hope will be followed.

Very truly yours,

W. L. SUTTON.

---

TO THE MEDICAL PROFESSION IN TENNESSEE AND KENTUCKY.

It is known to you that the American Medical Association is anxious to be supplied with correct and reliable histories of Epidemics prevailing in different portions of the Union. For this purpose it has divided the country into districts and appointed Committees to investigate and report on the epidemics of each. To enable these committees to discharge their duties to the best advantage, they are to continue for five years.

It will be seen at once, that if the physicians of the United States will set to work, record faithfully their experience, and at the end of each year transmit to the appropriate committee the result of their observations, we shall have a history of Epidemics greatly more reliable and valuable, and embracing a range of country vastly more extensive than our profession has ever seen.

Is it necessary to argue this point? Is it needful to say that this great object can only be accomplished through contributions from many individuals? Is it requisite to say to each individual physician, that he is personally called upon to furnish any facts which may be within his knowledge in aid of this great enterprise? It is hoped not.

The committee appointed for the States of Tennessee and Kentucky earnestly desire *carefully observed facts* upon any subject connected with Epidemics; but would suggest *particular* attention to the following heads:

Causes supposed to have given rise to the Epidemic.

Causes which favored its spread.

Causes which retarded its progress.

Prophylactics.

Age, sex, color, employment, diet, and habits as to exposure and temperance of those most liable.

Time and extent of its prevalence.

Prominent symptoms during its different stages.

Proportional mortality—*Post mortem appearances.*

Treatment.

Medical topography, including the nature and geological characters of the soil—character and average temperature of the springs and wells.

Meteorological observations—mean monthly and annual temperature, weight and moisture of the atmosphere, &c., &c.

It is considered important that as many cases treated should be reduced to tabular form as possible—a form for which is annexed—and returned with the reports.

As the members of the committees are widely separated, to enable them to consult each other, it is very important that reports be made by the 15th July annually to some one of them.

Wm. L. Sutton, M. D., Georgetown, Ky.
Asbury Evans, M. D., Covington, "
Frank A. Ramsey, M. D., Knoxville, Tenn.
Thomas Lipscomb, M. D., Shelbyville, "
E. B. Haskins, M. D., Clarksville, "
} *Committee.*

| | | NO. ATTENDED. | | | | | | | NO. DIED. | | | | | | | |
|---|---|---|---|---|---|---|---|---|---|---|---|---|---|---|---|---|
| | | Under 15 | | 15 to 40. | | Over 40. | | | Under 15 | | 15 to 40 | | Over 40. | | | |
| Diseases. | Color. | M. | F. | M. | F. | M. | F. | Total. | M | F. | M. | F. | M. | F | Total. | Avr. time of Attend. |
| Cholera, | W | | | | | | | | | | | | | | | |
| | B. | | | | | | | | | | | | | | | |
| Cholera Infantum, | W. | | | | | | | | | | | | | | | |
| | B. | | | | | | | | | | | | | | | |
| Diarrhea, | W. | | | | | | | | | | | | | | | |
| | B. | | | | | | | | | | | | | | | |
| Dysentery. | W. | | | | | | | | | | | | | | | |
| | B. | | | | | | | | | | | | | | | |
| Erysipelas, | W. | | | | | | | | | | | | | | | |
| | B. | | | | | | | | | | | | | | | |
| Fever, Bilious | W. | | | | | | | | | | | | | | | |
| | B. | | | | | | | | | | | | | | | |
| Typhoid | W. | | | | | | | | | | | | | | | |
| | B. | | | | | | | | | | | | | | | |
| Scarlet, | W. | | | | | | | | | | | | | | | |
| | B. | | | | | | | | | | | | | | | |
| Hooping cough, | W. | | | | | | | | | | | | | | | |
| | B. | | | | | | | | | | | | | | | |
| Influenza, | W. | | | | | | | | | | | | | | | |
| | B. | | | | | | | | | | | | | | | |
| Measles, | W. | | | | | | | | | | | | | | | |
| | B. | | | | | | | | | | | | | | | |
| Small-pox, | W. | | | | | | | | | | | | | | | |
| | B. | | | | | | | | | | | | | | | |
| | W. | | | | | | | | | | | | | | | |
| | B. | | | | | | | | | | | | | | | |
| | W. | | | | | | | | | | | | | | | |
| | B. | | | | | | | | | | | | | | | |

State how many of the above cases were seen secondarily,—in consultation or otherwise.

